# How ecovillages work: more-than-human understandings of *rentabilidad* in Mexican ecovillages

**DOI:** 10.1007/s11625-022-01162-7

**Published:** 2022-06-20

**Authors:** Olea Morris

**Affiliations:** Central European University, Quellenstraße 51-55, 1100 Vienna, Austria

**Keywords:** Ecovillages, Degrowth, More-than-human, Mexico

## Abstract

This article highlights the emergence of intentional communities known as ecovillages (*ecoaldeas*) in Mexico, exploring how humans seek to design sustainable futures in part by re-making rural livelihoods. Ecovillages are inherently speculative ventures, or as Burke and Arjona (2013) note, laboratories for alternative political ecologies, inviting—and indeed, necessitating—the reimagination of human lives with greater consideration for the natural world. In this sense, such communities might be understood as “exilic spaces” (O’Hearn and Grubačić 2016), in that they seek to build autonomous and self-sustaining agricultural, social, and economic systems while also reflecting a stance of resistance to neoliberal capitalist structures. At the same time, communities may also remain dependent on connections to broader regional or global markets in diverse and interconnected ways. Understanding ecovillages as diverse and emergent “worldings” (de la Cadena and Blaser 2018), I ask how these experimental social ventures reckon with their connections to the very systems they are positioned against. To trace out how communities negotiate this fragile space, this article is concerned with how ecovillagers spend their time at work—particularly when it comes to managing relationships with and between more-than-human beings. Drawing on participant observation with ecovillagers and more-than-human others they work with, I explore how the concept of “*rentabilidad*” (profitability) is differently constructed. To this end, I highlight ethnographic examples where *rentabilidad* is purposefully reconceptualized with more-than-human lives in mind; such a shift, I suggest, hinges on ecovillagers’ individualized relations with the beings they (imagine themselves) to care for.

## Introduction

In the wake of unprecedented ecological loss and social and political upheaval in recent years, the fragile relationships that sustain human experience on this planet are perhaps more visible now than ever before. With the understanding that these unfolding crises are “inseparable from the model of social life” made dominant by neoliberal capitalism and globalization (Escobar 2015:452), postdevelopment discourse has increasingly pointed to the need for decoupling development from economic growth (Escobar 2015; Acosta and Cajas-Guijarro 2020) and to instead seek out alternatives. To this end, conceptual engagement with the pluriverse—a world with the ontological capacity for multiple “worlds” (de la Cadena and Blaser 2018)—gestures to the possibility of a diverse “matrix of alternatives” (or alternatively, a “postdevelopment rainbow”), rather than a unilinear approach to sustainable development (Demaria and Kothari 2017; Akbulut et al. 2022). Ethnographic attention to how grassroots and localized alternatives to development are shaped by their geographic, historical, social, and epistemic contexts (Escobar 2015:456) has been instructive, gesturing to the ways that “communities, societies, and landscapes, especially those dominated by industrial capitalism,” might be re-imagined, unmade, or rebuilt (Brightman and Lewis 2017:2). By the same token, I argue, engagement with the pluriverse also entails revisiting the logical premises that have underpinned past approaches to sustainable development to develop a more capacious sensibility of “what works” in practice.

In keeping with the theme of this Special Feature on the “Pluriverse in Practice” (Akbulut et al. 2022), this article discusses ecovillage communities (*ecoaldeas*) in Mexico to explore how different communities carve out spaces of possibility and flourish from within contexts shaped by global flows of extractive capitalism. Situated at the convergence of several transition discourses, including co-housing, commons thinking, and degrowth (Lockyer 2017), ecovillages are broadly defined as communities that work to consciously pursue economic, social, and ecological sustainability (Jackson 1998) through the “harmless integration of human activities into the environment in a way…[that] is able to continue into the indefinite future” (Kasper 2008). As López and Silva Prada (2015) observe, ecovillages seek to respond to social issues understood to stem from “the environmental crisis and/or the loss of meaning of the development model of contemporary capitalism,” (2) seeking to enact change by living it. Ecovillages do not follow a particular blueprint or plan, but instead co-construct community values and practices in place (Meijering et al. 2007; Dawson 2013; Dias et al. 2017). These practices might include collectively growing food without pesticides or conventional fertilizers, building homes and structures with locally sourced, renewable materials (“natural building”), or reducing energy consumption (i.e. “off-grid” or carbon–neutral lifestyles) (e.g. Kirby 2003; Meijering et al. 2007; Litfin 2012, 2014; Pires and Lima 2013). As such, the ecovillage model has inspired academic attention to how it might be “scaled up” or imported to new contexts (Litfin 2009; LeVasseur 2013; Singh et al. 2019; Temesgen 2020).

The question posed in the title of this article—“How do ecovillages work?”—is directed towards this ostensible tension between autonomy and “radical interdependence” (Escobar 2018), asking how ecovillage residents pursue alternative livelihoods while negotiating interconnectivities with the same broader economic and social systems they might be positioned against. Ecovillages are far from being “islands of sustainability” (Andreas 2013; LeVasseur 2013), but rather remain deeply interdependent with the world beyond their boundaries, both socially and economically (Dawson 2006; Burke and Arjona 2013; Farkas 2017); as such, residents “simultaneously live in the ecovillage and the larger society” (Ergas 2010:33). As Jonathan Dawson, former president of the Global Ecovillage Network (GEN), has observed, “ecovillage enterprises, in common with all others in the capitalist economy, depend on a culture of consumerism that far outstrips the meeting of basic needs” (2006:56–57). Moreover, Baker (2013) suggests that these lingering connections to broader systems can work to complicate or ultimately sunder communities: “after all, the factors that these community founders are citing—lending policies of financial institutions, property values, and zoning regulations—can be linked directly to capitalist processes and dynamics” (293). These considerations reveal the ways that the performance of alternative livelihoods requires substantial negotiation between sustainable visions and context or circumstance.

Understanding ecovillages as kinds of “exilic spaces,” or spaces on the margins of social and economic life “in which people attempt to escape from capitalist relations and processes” (O’Hearn and Grubačić 2016:148), I ask how ecovillage communities renegotiate their relationships to the broader social and economic systems in which they are entangled (Dawson 2013). Drawing on ethnographic research conducted in two ecovillage communities in two states in Mexico, I call attention to instances in which interlocutors engaged with the concept of *rentabilidad* (profitability) in the contexts of the regenerative agricultural systems and community spaces they construct. Building on understandings of relational value (Saxena et al. 2018; West et al. 2018), I problematize the normative associations between profitability on the one hand and the perceived viability of alternative community models on the other. Undoing these connections involves attending to the socially constructed dimensions of profitability, and the different ways that profit—understood here as the return of advantages or benefits relative to the time, labor, attention, or resources invested—is reconfigured, particularly in relation to more-than-human lives.

This article is structured as follows. I first develop a theoretical understanding of *rentabilidad* through a more-than-human lens, explaining how this idea complicates existing approaches to economic sustainability. I then trace this argument through the particular contexts in which ecovillage communities in Mexico are situated, identifying three ways in which relationships with more-than-human others influence the ways that ecovillages “work” (construed both as concrete practices, as well as their perceived functionality or viability). Finally, I relate these examples back to the broader discussion of the pluriverse-in-practice, touching on how ecovillage communities collectively reframe practices in relation to broader values.

## More-than-human understandings of *rentabilidad*

While economic sustainability has long been enshrined as a key dimension of sustainable development discourse (Purvis et al. 2019; Spangenberg 2005; Zhong et al. 2021), scholars have highlighted how a persistent conceptual ambiguity has complicated attempts at putting it into practice (Norton and Toman 1997; Hinton 2020). Owing partly to this undertheorization, conventional economic logic continues to underpin the way sustainability is framed: in particular, the assumption that markets are inherently driven by profitable growth (Hinton 2020). As Spangenberg (2005) notes, “continuous and indefinitely (or at least long-term) sustained growth is—often implicitly—assumed to be part of sustainable development of the economy” in mainstream policy debates (49). This framing has a distinct influence on the way sustainable development is pursued. Based on the unquestioned understanding that “economic vitality” is a necessary precondition for increasing living standards or fostering social improvements (Purvis et al. 2019; Rainey et al. 2003; Spangenberg 2005), economic growth (and profit, as an analog) has come to be framed as tools for furthering both social and ecological sustainability objectives (Rainey et al. 2003). The understanding that “people, planet, and profit” are commensurate and mutually achievable objectives has been further reproduced through the emergence so-called “hybrid organizations” or “social enterprises” (SEs): business models that have sought to realign “profit with social impact” and environmental responsibility (Bocken and Short 2016; Alberti and Garrido 2017).

The unexamined premise that profitable growth is a necessary condition for achieving sustainability outcomes is problematic for several reasons. First, scholars have pointed out that enterprises oriented towards profit maximization and capital accumulation simultaneously drive environmental damage and social inequality alongside economic growth (Parrique et al. 2019; Hinton 2020), revealing the inherent paradoxes of using the former in the service of addressing the latter. Second, scholars have increasingly demonstrated how conceptions of value encoded in measures of profit are socially constructed and relational (Graeber 2001; Retsikas and Marsden 2018). For this reason, reframing profit as an objective that can be attained through the adoption of more sustainable (or what are increasingly described as “circular”) practices still fails to offer an explicit critique of the “value-creation logic” (Schaltegger et al. 2016) that underlies these conventional understandings of what profit is or how it might be attained (Jackson 2009). Finally, scholars have increasingly called attention to possibilities for “living well” that do not hinge on profit or economic growth, examining diverse expressions of communitarian and grassroots approaches to sustainable livelihoods (Kothari 2014; Kothari et al. 2014), or speculating how a downshift to not-for-profit business models could facilitate postgrowth transitions (Hinton 2020). In short, conflating profitability with viability consigns us to a narrow interpretation of “what works” and what does not, limiting the scope of alternative socioeconomic models considered.

Though profitability is a contested term (Child 1998), I use it here to refer to a constellation of labor, value, and time, expended in such a way that it produces a gainful yield (as perceived by community residents themselves). Although profit is normatively associated with capital, conversations with ecovillage residents revealed the ways that this concept was reconfigured to account for ecological and social, as well as economic, abundance (Jackson 1998). This analytical focus was particularly inspired by a conversation with an ecovillage resident named Katrina, where she described her vision for the future of her community: “the point is to be *autónoma* (autonomous). That’s the idea—liberty. Not to *just* be *sustentable* (sustainable), or *autosustentable* (self-sustainable), like making money through workshops, eating our own food…but a project also has to be *rentable* (profitable).” When encouraged to explain further, I was surprised by her answer: “not just making money, of course, but also politically *rentable*… socially *rentable*. To help to make things better, but to still live simply.” This exchange gestured to the subtle ways that seemingly neutral economic language could be reframed and suffused with new meaning to align with community objectives. Neither of the communities discussed here could be considered conventionally “profitable” (in the sense of earning surplus capital from selling agricultural products, holding workshops, or hosting visitors). Instead, Katrina’s reflection suggested the possibility for fundamentally remaking the rubric by which community successes were evaluated: not by “profit” as a standardized unit of value, but rather localized and collectively negotiated sensibilities of *rentabilidad*.

In focusing on these qualitative shades of *rentabilidad*, I engage with ongoing streams of discourse from environmental studies, anthropology, and ecological economics that have sought to understand how alternative values are relationally constituted between humans and more-than-human others (c.f. Chan et al. 2018; Saxena et al. 2018; Himes and Muraca 2018; West et al. 2018). As Saxena et al. (2018) argue, greater sensitivity to more-than-human actors in qualitative research has revealed the complex ways humans inscribe meaning on more-than-human bodies through practices of labor and care, and how these relationships co-constitute one another (Kirksey and Helmreich 2010; Krzywoszynska 2016; van Dooren et al. 2016). Such an approach considers more-than-human beings as creative, agential forces, or rather “as ends in themselves, and not just means to human ends” (Latour 1998, cited in Kasper 2008:12).

This relational value perspective emphasizes ways that humans make meaning and assign value to their environment and other living beings in ways that extend beyond instrumentality (Lukka 1990; Haraway 2016; Himes and Muraca 2018), understandings which are not only forged through “labor and productive relations” (Saxena et al. 2018:56), but rather are “reflective and expressive of care, identity, belonging and responsibility, and congruent with notions of what it means to live a ‘good life’ (West et al. 2018:35). Engagement with different practices of alternative agriculture—including biodynamic agriculture, permaculture, and agroecology—becomes ways of “enacting contestations in the fields”, and in doing so performs “alternative rural realit[ies] of mutualism and abundance” (Münster 2018). Turning attention to the ways that ecovillage residents design and maintain networks of multispecies relationships reflects underlying values of particular communities, but also how these values are negotiated by residents through their daily practices (Krzywoszynska 2016; de la Bellacasa 2017).

## Methodology and research sites

This research draws on participant observation and ethnographic fieldwork carried out over approximately 13 months in ecovillages throughout Mexico between June 2018 and November 2019. For purposes of salient comparison, I focus on two communities: Aldea Ceiba in the state of Yucatán, and Rancho Bosque Rancho Escuela (Rancho Bosque Ranch School) in Veracruz.^1^ I spent approximately 4 months divided into two periods during different seasons, serving as a volunteer in both communities. This arrangement of working (5–6 h per day) in exchange for accommodation allowed me to live in residence with both communities and develop familiarity with interlocutors. This role also allowed me to work with and alongside residents in roles that ranged from preparing communal meals, harvesting crops, cleaning stables, or tending to compost piles alongside different residents. Moving between communities allowed me to be present for important community events, or to observe differences in priorities and work practices at different times of the year.

Aldea Ceiba community was founded in 2015 outside of a small community in central Yucatán by a group of approximately 9 residents (known as *semillas*, or “seeds,”) and a continuously rotating community of volunteers, visitors and friends. The tropical climate permits the cultivation of diverse agroforestry systems, informed by regenerative agricultural practices such as permaculture or syntropic agriculture, an approach to cultivating agroforestry systems by mimicking forest succession cycles (Andrade et al. 2020). The community describes itself as a center for the “interchange of knowledges,” (*intercambio de saberes*), and in addition to experimenting with local agricultural techniques and eco-technologies (including rocket stoves, bike-powered appliances, and solar panels), they also held workshops and community gatherings (*tertulios*) on topics such as native bee conservation or recuperating traditional *milpa* farming systems. Residents often earn an income from their own projects within the community, although incomes are partially pooled and redistributed to particular members as needed. A beekeeper in Aldea Ceiba, for instance, might keep the proceeds from holding paid workshops on bee care, while honey sales might be directed back to the community.

The second community, Rancho Bosque, lies adjacent to a protected cloud forest on the outskirts of a large urban center in Veracruz and comprises several pastures and shade-grown orchard spaces as well as patches of native forest. The community ranges in size from five to twenty people at any one time, composed of permanent residents, apprentices who live in the community for several years as part of a live-in educational program, and foreign volunteers. Rancho Bosque predominantly practices biodynamic agriculture, a form of organic agriculture with elements of spirituality and folk practices that has roots in Central Europe (Paull 2011). A printed vinyl sign, hung at the entrance to the community’s land, summarizes the key message the founders hoped to impart to the young apprentices and visiting students:

“You can and should be proud, to practice the most important profession that exists. To work the land, to raise animals, to take care of the environment, the forest, the soil, the water. Without you, without agriculture, there would be no known development, culture would not have developed, and without this no known civilization. Be proud of yourselves!”

The ideological emphasis on self-sufficiency is connected deeply to their work with livestock, although they also maintain gardens, grow shade-grown coffee and macadamias, and have a small dairy and bakery. Although occasionally animals are sold to neighbors or other local farmers, the primary focus of caring for livestock is in developing practical education programs for young “apprentices” who live on site over the course of several years to learn how to manage their own holistic agriculture projects.

On the surface, these communities differ substantially from one another—located in distinct ecological and geographic contexts, they have different and at times contradictory approaches to issues ranging from agriculture to styles of conflict resolution. At the same time, both communities share similar concerns for the future livability of the planet brought about by consumption-driven capitalism, pollution, deforestation and loss of biodiversity. Both communities practice forms of subsistence organic agriculture, rely on forms of renewable energy (solar and biogas), build homes and communal spaces with local or biodegradable materials, and seek to reduce or re-localize consumption to the greatest extent possible.

I carried out in-depth participant observation and semi-structured interviews (~ 79) with residents, visitors and volunteers, asking participants to reflect on their roles in the community and how they went about their daily routines. While residing in these communities, I attended and documented evening lectures, cultural events, and workshops on topics such as soil care, bee care and holistic veterinary practice. In particular, I sought out opportunities where human residents interfaced with the animals, plants, insects, and other species present in their communities by following patterns of daily work: helping with tasks such as checking beehives, cleaning stables, cultivating and maintaining gardens, or planning the rotation of crops and livestock in the ecovillage landscapes, providing rich opportunities for understanding how residents conceptualized value in relation to their work with more-than-human others.^2^ Fieldwork notes and informal encounters and conversations also contribute to the understandings discussed in this article.

## Resistance in place: situating Mexican ecovillages

The emergence and popularization of the ecovillage community model in Mexico must be understood in relation to increasingly neoliberal and industrialized approaches to rural development in Mexico over the last century. Following the Mexican Revolution of 1917, over one-half of Mexico’s arable land was redistributed to indigenous communities and groups of smallholders, who were given usufruct rights to communally held lands known as *ejidos*.^3^ Over time, however, smallholder agriculture came to be compared disfavorably to the “efficient, large-scale modern farms” that were ushered in by the Green Revolution and spending on rural infrastructure increased greatly in the early 1940s (de Janvry 1981; Frye 1994). State support, subsidies, and “high-yield seeds” were increasingly directed to these larger agricultural enterprises (de Janvry 1981:124) in an effort to spur economic development through exports. In 1992, an amendment to the Mexican constitution permitted the private sale of *ejidal* lands and helped pave the way for the adoption of the North American Free Trade Agreement (NAFTA) in 1994, effectively ending state support for the *ejido* (Sonnenfeld 1992; Frye 1994; Yetman 2000; Perramond 2005). Each of these developments reflects pieces of a common central narrative shaping rural development policy in Mexico in the last decades: namely, the conversion of “informal” smallholder economies into an agricultural sector through industrialization and privatization.

Self-sufficiency and autonomy have been common conceptual threads of the social and environmental movements premised on resistance to these policy developments. Carruthers (1996, 1997) discusses how indigenous and environmentalist movements became linked in their mutual resistance to structural policies that prioritized economic development at great cost to the socioeconomically vulnerable and the environment. This has perhaps been most notably exemplified by the mobilization of the Zapatista Army of National Liberation (EZLN), an autonomous movement that emphasized indigenous rights and food sovereignty the day that NAFTA came into effect (Naylor 2012; Hernández et al. 2020). As Barkin (2006) notes, these developments produced a range of grassroots alternative development models, with more communities “forging alternatives that allow them greater autonomy” and “experimenting with new productive combinations that allow them to strengthen their communities” (137). In such a way, traditional practices of agriculture, landscape management, and species care have also become forms of resistance in and of themselves, as alliances between indigenous rights and environmentalist groups both “center on the effort to preserve and defend traditional ecological knowledge” (Carruthers 1997:259). Communities like Rancho Bosque and Aldea Ceiba locate their broader community objectives at the intersection of these discursive threads of resistance, albeit in different ways. While founders at Rancho Bosque hoped to combat the decline of smallholder agriculture by providing training and resources to local youth interested in managing their own holistic farms and ranches, Aldea Ceiba residents collaborated closely with Maya neighbors in maintaining the health and biodiversity in their agricultural systems.

Similar to other ecovillage projects that have been documented elsewhere, many residents come from largely upper-middle class and urban environments (Farkas 2017). Long-term visitors and volunteers to Rancho Bosque and Aldea Ceiba are a mix of foreign nationals from North America, Europe, and Latin America as well as Mexican nationals (largely from other urban centers). The founder of the Rancho Bosque community was raised in Europe, while Aldea Ceiba was composed largely of a group from Mexico City and surrounding environs. Foreign ecovillage founders benefitted from access to foreign passports, pensions, and healthcare plans, while enjoying a relatively lower cost of living on the strength of foreign currencies. Although foreigners are not able to own *ejido* land outright, informal contractual and/or good faith agreements with Mexican residents extend the circle of de facto stakeholders in the community. These arrangements rely heavily on trust amongst community members, and can be tenuous or unstable.^4^ At times, these differences were recognized and explicitly worked against. In Aldea Ceiba, for example, the amount of requested donations for volunteers from Mexico and other Latin American countries was lowered relative to that of other foreign volunteers (largely composed of travelers from elsewhere in North America or Europe) due to recognition of the socioeconomic imbalance between visitors and volunteers from differing backgrounds.

Despite the fact that community residents are oriented towards the goal of self-sufficiency, both communities retain dependencies on the “outside world” to support their community’s activities. Both Rancho Bosque and Aldea Ceiba rely heavily on the labor of volunteers and visitors in caring for animals, managing cultivated areas, leading workshops or preparing artistic or cultural events, cooking communal meals, or other key tasks. Both communities advertise their projects on online portals such as WWOOF (Worldwide Opportunities on Organic Farms) or Workaway in search of volunteers, many of whom are foreign travelers with interests in environmental or social activism and/or sustainable agriculture (what Velázquez Castro 2018 calls “voluntourists''). Many foreign volunteers contribute labor in exchange for modest accommodation and meals, sometimes contributing a nominal fee for expenses incurred (for example, additional food or transportation). Additionally, several residents in Rancho Bosque and Aldea Ceiba from Mexico received funds from a new nation-wide social program known as *Jóvenes Construyendo El Futuro* (Youth Building the Future), which allows young people (18–29) who are not working or studying to ally themselves to participate in various forms of employment training with different companies or civil society organizations. Together, each of these sources of funding and income was instrumental to supporting each community’s fluctuating population of volunteers and visitors, and was an important supplement to subsistence-oriented agricultural practices.

Ecovillage residents in both communities rely heavily on their cultivation of various plants, animals, and insects, both for self-consumption and for outside sale. Rancho Bosque sells a variety of products in a small storefront at the edge of their property and at local markets in the nearby capital of Xalapa; these include value-added products like cheese and dairy products or baked pastries, as well as specialty staples such as coffee, nuts, or honey. At Aldea Ceiba, different kinds of honey as well as specialty products like medicinal tinctures and locally produced handicrafts were sold in a small shop located on site, or distributed to specialty shops in nearby cities or through residents’ social networks. The relative lack of a local market for ecologically produced (and consequently, more expensive) specialty products is in part a function of the location in rural areas in which ecovillage communities tend to be located, which are by and large, areas where local consumers are unlikely to be able to afford them, complicating attempts at integration into local markets.

While caring for more-than-human others is and has long been a part of rural livelihoods, ecovillage communities differ from other kinds of traditional or agrarian communities in two primary ways. First, new ecovillages are largely products of intentional design, and as such seek to self-consciously organize social and ecological relationships in harmonious ways. Agroecological systems are often premeditatively designed with the goal of creating “self-sustaining” systems, which are maintained in ways that are seen to mimic “ecological” or “natural” processes. Second, many ecovillage residents (especially those from largely urban environments) have little or no prior practical experience in agriculture or the ecological contexts in which these communities are founded. As such, the process of maintaining agroecosystems implies a highly experimental and collaborative approach in their design, relying on relationships with local experts, practitioners, and neighboring communities. In this way, ecovillage residents are not necessarily novel in their approach to agriculture, but rather exhibit novel ways of relating to or engaging with forms of indigenous or traditional land management practices and agricultural strategies in the service of constructing new livelihoods.

The importance of these social relationships became apparent in Aldea Ceiba’s response to the dual crises affecting communities in Yucatán in early 2020: devastating flooding caused by tropical storm San Cristóbal, and the COVID-19 pandemic both damaged crops and prevented workers from taking up regular means of employment, largely in the tourism sector, in nearby coastal cities. In response, two residents of the Aldea Ceiba community founded an initiative to provide resources for local families to cultivate traditional home gardens for self-consumption, funded in large part from donations of past volunteers solicited through online social networks, as well as regional organizations with whom they had previously collaborated. The project, which aims to address economic and food insecurity, draws on the already-embedded relationships ecovillage residents have built within neighboring communities by pooling resources to enable local families to develop home-scale cultivation of traditional crops. In the words of the project leaders, “we have woven intercultural relationships based on respect and cooperation with various families, which today allows us to have the conditions to develop this new project that links food sovereignty, the regeneration of community relations, along with the work of the land and the flourishing of women as leaders for the cultural transformation that we need around the world today.”

In the sections that follow, I build on this contextualization by highlighting the ways that ecovillage residents construct and negotiate understandings of value in relation to their work with more-than-human others. Using *rentabilidad* as an orienting concept to capture processes of value construction, I explore how residents of Aldea Ceiba and Rancho Bosque mediate and refine community goals in relation to more-than-human entities through three dimensions: alternative relations of scale, alternative values, and alternative temporalities.

## Alternative relations of scale

One of the ways in which ecovillage residents renegotiate understandings of profitability is by reconfiguring the scale of their activities around more-than-human lives. Past approaches to sustainable community development have been often oriented towards transforming rural communities into adaptable, networked market actors: “for rural communities to succeed in the global economy,” it was reasoned, “they must be able to compete not only with other rural communities both at home or abroad but also with urban areas” (Rainey et al. 2003). As Tsing (2015) argues, framing “scalability” thusly requires homogenization, in that it requires “a project to change scales smoothly without any change in project frames,” and as such, “that project elements be oblivious to the indeterminacies of encounter” (Tsing 2015: 38). The imperative to scale up is often incongruous with the objectives of communities like Rancho Bosque and Aldea Ceiba, however, which seek to scale back consumption practices and instead resituate livelihoods within local ecologies. Rather than working to build economies of scale that also happen to be “ecological,” residents instead reframe relationships with external sources around community residents—both human and not.

Residents at Rancho Bosque and Aldea Ceiba both expressed that a key goal was to recenter consumption practices around what the community was able to produce, rather than vice versa. In both communities, raising livestock or cultivating gardens was primarily understood to be for consumption within the community rather than for sale (as an apprentice from Rancho Bosque remarked proudly, “we don’t grow anything we don’t also eat ourselves.” While some residents acknowledged that the goal of complete “self-sustainability” (*autosustentabilidad*) might not be wholly attainable, the conceptual benchmark was frequently invoked nonetheless in community discussions about consumption habits. In the dining room of Rancho Bosque hung a sign with the title “Achieving a society without war, fear, hunger, [and] with better education—what do we have to do to bring us closer to our dreams?” The list of key principles below urged residents to reconsider their consumption patterns: “don’t be blackmailed (*chantajeando*) by the pressure to buy a certain type of clothes, a phone, a drink, or some junk food…the true happiness will be had by the one who has produced the items not necessary for your life.”

While not all community members share the same ascetic zeal (and occasionally indulge in said “junk food” or alcohol purchased on supply runs in town), orientation towards the broader goal of self-sufficiency has still inspired some community-wide changes in consumption patterns. Manu, a longtime resident of Aldea Ceiba, reflected how the community’s dietary patterns had shifted in relation to the challenges of growing particular crops (leafy greens or root crops in particular) in the nutrient-poor soils of the Yucatec forest. “Now we experiment a lot more with native seeds and plants, because that’s what we have—the things we’re meant to eat in this environment.” Among the community’s most successful experiments (perhaps unsurprisingly) were recipes shared by neighbors or inspired by staples of Mayan cuisine, such as *balché*, a fermented alcoholic drink made with honey, or bread made with the flour from the nut of a *ramón* tree (*Brosimum alicastrum*, or *yaxox* in Maya). The immense value of this local knowledge could not be underestimated, one of the founders of the community emphasized to me: “we nurture those relationships [with our neighbors] in good faith, because without them we wouldn’t be here.”

While volunteers and paying visitors are essential in helping ecovillage communities run smoothly, permanent residents often prioritize the integrity of ecological systems over accepting more paying guests and volunteers, expressing these limits in terms of more-than-human actors. In Aldea Ceiba, one resident who was tasked with giving tours to newcomers, often paused at a shady grove of mangoes, located off the forested trail to the meditation platform. “We love having volunteers, but sometimes it is too much. We are surrounded by water, but all of it here in Yucatán, it’s underground,” said the resident, referring to the below-ground channels and pools (*cenotes*) formed in the karstic limestone bedrock of the region. “These mangoes are our guide for when to stop accepting new people. When we have too many people—thirty people, or more—especially in the dry season, these trees will start to droop,” he told the group, emphasizing his point by frowning and mimicking limp, unwatered leaves with his arms. Likewise, Rancho Bosque paused their volunteer program for several months following the death of several sheep in their herd, even declining offers from paying visitors. The resident in charge of managing livestock rotations explained that the decision was based on the perception that the general health of the flock always seemed to take a dip when new volunteers were present. Volunteers not only required training and consideration, but also brought unpredictable “energies” that had the potential to disrupt the subtle rhythms of the flock. The cascading effects perceived to stem from this lack of attunement to animal energies (“an unhappy animal is going to transfer that energy to its milk, which then affects the cheese that comes from that milk,” he explained) could have significant consequences, both for the integrity of the products they hoped to sell as well as the survival of the flock itself.

Engaging with more-than-human actors in particular ways also opens up and facilitates particular avenues of financial support. Both Rancho Bosque and Aldea Ceiba were registered as *Asociaciones Civiles* (A.C.), a particular kind of registered civil society, not-for-profit association. Registration as an A.C. allows ecovillage communities to formalize aspects of their work, including the foundation of educational programs in regenerative agriculture or sustainable pasture management, or organizing and promoting cultural events. Additionally, such affiliation allows ecovillage communities to legitimate external forms of support, for example, from governmental funds for the development of regional culture, development institutions, or private individuals. In particular, Rancho Bosque was supported by a German development fund focused on promoting biodynamic agriculture, particularly in developing countries. Similarly, the work of two community members of Aldea Ceiba on the topic of native bird conservation brought their community into contact with local conservationists, who provided resources for initiating a youth club in the local community dedicated to birdwatching and engaging in waste management and conservation activities. Ultimately, these relationships culminated in a program through which selected students from the community group were invited to the United States to participate in an outreach program offered by a renowned biological research institution. In this way, residents assembled networks of external collaborators and supporters through their work with particular kinds of species or agricultural practices, using these associations to root in place rather than expand outwards.

## Alternative values

By alternative values, I refer to the ways that ecovillage residents seek to reframe community goals in relation to more-than-human lives. As Kasper (2008) observes, what distinguishes the ecovillage paradigm is “an expanded notion of ‘community,’ one that includes not only people, but countless other species as well” (22). Through this acknowledgement of self in relation to “interdependent parts and processes” of the living systems which support the community, (Kasper 2008:22) ecovillage residents conceptualize the value of their work with other species in ways that transcend strictly economic benefit.

Residents in different communities identified strongly with their roles working with particular species. Although residents in both communities had broad knowledge across various domains of community agricultural operations, individuals gravitated towards particular roles either through self-selection (Aldea Ceiba), or through rotating assignments (Rancho Bosque). Relationships formed in these daily roles contributed to the formation of particularized kinds of knowledge that could have only been generated through regular experiences. Relative to this full-time schedule with few (if any) days of vacation, the cash with which residents were provisioned monthly was paltry. When asked about this discrepancy, however, I was surprised to hear that many residents felt the exchange was fair. “I’m not working for [the farm manager], and I’m not necessarily working for the salary,” said one young farmhand, who had lived in the community for nearly a year caring for their herd of sheep. Instead, this resident expressed a deep commitment to the land and his flock, and highly valued the opportunity to gain experience in different ecological farming practices without assuming the financial risks of land ownership himself.

Relationships forged with particular more-than-human others draw residents into care relationships with still other species. As one shepherd told me of his work with the sheep, “I call myself a *pastor* (shepherd), but actually I am really engaged with *praticultura* (the management of grasslands),” referring to the fact that he spent most of his days not with the sheep, but rather managing the community’s parcels of pasture. This is to say that focusing on the health of sheep meant also attending to the sheep’s web of relationships with soil, parasites, and native vegetation. This practice of “nested cultivation”—caring for particular beings like grass, with the understanding that their care will have ancillary effects for other valued beings—shows how understanding the broader ecological and social role of individual animals or plants influences the way that work is undertaken.

In the Aldea Ceiba community, this was evidenced by their dual cultivation of several species of native bees as well as *Apis mellifera*, the European honey bee. Beekeepers in Aldea Ceiba community cultivated habitats for native bees both directly, in caring for and managing hives of particular species, and indirectly, by cultivating native vegetation pollinated by specialist bees or by actively conserving existing native bee nesting sites around their land. In doing so, community residents actively participated in the conservation of endangered native species, particularly those which were of cultural significance to the local communities. While both produce honey, *Apis mellifera* produced far more honey by weight per hive than did *Melipona beecheii*. Caring for *Apis mellifera* bees enabled Aldea Ceiba residents to care for *Melipona beecheii* bees, in that revenue generated from both workshops and the sale of bee products allow ecovillage residents to continue cultivating *Melipona beecheii* hives. The cultivation of native bees, however, transcended their utility as a species that produced a marketable product. Rather, cultivating *Melipona* bees allowed the community residents the ability to engage on a material level with local communities, particularly local beekeepers.

This engagement with native bees also implicates community members in the broader discourse of indigenous land tenure and dispossession, and the survival of cultural traditions. Because it is rare to encounter *Melipona beecheii* hives outside of human cultivation, the hives that Aldea Ceiba tends to are divisions from hives borrowed from their neighbors. At the same time, honey produced by the *Apis mellifera* bees was used to sustain the ongoing conservation work that the community had organized around native bees. Not only did income from the sale of *Apis mellifera* honey help to fund the construction of more apiaries for native bees, but *Apis* honey was also used as a way to feed native bee colonies during the wet season, when food stores are low.

These material relationships are underscored by the relationships that the community’s beekeepers construct and maintain with the hives they care for over time. One founding resident became the community’s beekeeper over time after encountering the native *Melipona* bee for the first time, developing an affinity for the “calm” species^5^ that seemed to mirror her own personality. Participating in external beekeeping conferences and workshops held at a local agroecology school allowed this resident to deepen their understanding of the differences in caring for native bees as opposed to the more widely known *Apis mellifera* species, and also to become conscious of the species’ precarious future.^6^ Mindful that any honey harvested from the *Melipona* bee represented a possible drain on the health or energy stores of the hive, the beekeepers only extracted small amounts of honey, using a hollow-tipped syringe to draw the honey out of the “pots” the bees stored it in, instead of slicing them open with hot knives. By respecting the work of the bees themselves, the resident reasoned, the bees would hopefully flourish to return the favor in kind.

## Alternative temporalities

A third angle through which ecovillage residents unmake conventional understandings of profitability is by unmaking relationships between time and value: what Kolinjivadi et al. (2020) call alternative “escape” temporalities, or time scales that resist capitalism through incongruity. In capitalist systems, time is conceived of as “predictable, homogenous, linear… and endlessly unfolding” (Kolinjivadi et al. 2020; Koretskaya and Feola 2020:306), and this understanding is performed in conventional agrienterprises in a variety of ways: accelerating food production, manipulating (or eliminating) seasonality, or privileging “modern” or technologically intensive techniques (Castree 2009; Koretskaya and Feola 2020). On the other hand, scholars have documented how grassroots movements and local communities construct counternarratives, critiquing and remaking relationships between value and the passage of time (Kolinjivadi et al. 2020; Bastian 2019). At Rancho Bosque and Aldea Ceiba, evidence of these re-negotiated understandings is influenced by relationships with more-than-human residents in two senses: first, by synchronizing patterns of work to the life-rhythms of more-than-human others, and second, by recasting the value of expended labor-time in terms of net benefit to the community as a holistic system (rather than in the service of particular profit-seeking activities).

In seeking to contest what they framed as a cultural loss of connection with land, ecovillage residents consciously adopted practices and styles of working that took into account seasonality and ecological cycles. Taking time to work “properly” with the animals and plants they cultivate involves organizing work activities around more-than-human timescales. This was evidenced in part by the rotational grazing system implemented by Rancho Bosque, through which the movements of animals through particular pastures was carefully orchestrated so that no animal re-entered the same parcel twice in a month span. Such rotations reflected both the life cycle of pasture grass, and the time it took to reach “optimal” height for re-grazing, as well as the life cycle of intestinal parasites present in recently browsed pastures. While such rotational practices demand more time and direct management by shepherds, such systems were seen as the only logical, “profitable” move—in that neglecting this balance could permanently degrade soils.

Working on smaller scales—with fewer hands and resources than conventional operations, for instance—necessitates much greater individual investments of labor and time. However, many residents reframed these (often uncompensated) investments of time as the opportunity cost for the multiplied benefits of living in community. One evening I found a young shepherd named Alejandro in the *panadería* (bakery), where he had been scraping bits of meat from the skull of a slaughtered ewe while chatting with a friend. Earlier in the day, a visitor to the community’s storefront had inquired about the price of purchasing mutton, and I posed the question to Alejandro—surely, the amount of time and attention devoted to processing the carcass would be reflected in the meat’s price at market? He grinned wryly, and explained it was quite the opposite: slaughtering the ewe had taken nearly all day, and he was only completing the task by evening firelight. Without even accounting for the time he had devoted to all that managing the sheep flock entailed (cleaning stables, cutting and drying forage, or providing medical care), there would be no point to sell the meat. Still, he considered his work more than worthwhile: “What we have here is enough for us to eat well,” he said, referring to other residents, visitors, and volunteers. “For my time, it’s worth it for us, but not to sell it.”

Closer attention to factors such as seasonality and more-than-human lifetimes resulted in a diversification of economic practices, as profit was generally understood to derive from the whole of community landscapes and more-than-human residents in concert, rather than the particular components or beings comprising those systems. At Rancho Bosque, coffee and macadamia nuts were harvested intermittently throughout their fruiting cycles, while young pigs or lambs were butchered and sold as individuals came to maturity or when herd populations began to outstrip available pasture space. In community meetings at Aldea Ceiba, residents were encouraged to gather particular kinds of native seeds or fruits at their leisure as they came into season: tufts of cotton-like fiber from the seed pods of the *ceiba* tree (*Ceiba pentandra*) that often blanketed the ground in March, or the edible seeds of the *pich* (*Enterolobium cyclocarpum*) or *pixoy* (*Guazuma ulmifolia*) trees.^7^ Because processing seeds was extremely time-intensive but required little concentration, this work was often folded into (or became an excuse for) leisure time: small groups of residents inevitably formed in the communal *palapa* around piles of hulled seedpods, especially in the midday heat of the dry season. Working alone, the work of prying small seeds from their hulls was considered hardly worth doing because of the time required to produce an amount of any significance. As a collectively performed pastime, however, the work became worthwhile.

One particular experience working in Rancho Bosque’s garden spaces reflected the differences in how residents understood the expenditure of their time relative to resultant benefits. After preparing beds for the transplantation of seedlings, the gardener whom I was working alongside enlisted the help of two apprentice-shepherds and myself to “dynamize” (*dinamizar*) the water we would supply the plants with after planting.^8^ For the next hour, myself and three residents would stand huddled around the blue plastic rain barrel, stirring the water in a clockwise motion with a long, broad stick. As soon as the water began to swirl around the edge of the barrel, creating a tunnel that extended nearly to the bottom, the stirrer would heave the stick back and begin stirring in the opposite direction. The force necessary to whip the water one way and then another was exceedingly difficult to sustain for any great length of time, and we passed the stick between us every few minutes.

As we stirred the water, with the goal of “bringing downwards” the energetic forces from the air into the water that would be used to germinate new seedlings, I considered briefly how such a task would be rendered absurd in an industrialized agricultural system, where “time is money.” Under such assumptions, a task that absorbed the attention of four workers for the course of an hour (but with ostensibly little change in the result) could hardly have been considered productive. At the end of the hour, when the trail of water began to slow, he cupped a handful of the water out of the bucket and observed it. “The water feels softer now,” he says, explaining that the change in color and texture indicated that the bodies of microorganisms in the water had been broken up by the force of the vortex. The water was sufficiently energized now, he explained, and ready for application on the new seedlings to be planted in accordance with the new moon that would be occurring that month. When prompted, the gardener explained the value of taking time for caring about seemingly mundane details was one of the privileges of working in smaller scale agricultural systems: “we have time to bring the energy in, to care about the energy—we’re not trying to produce as much as we can.” The experience revealed how the performance of alternative relationships with time constituted a central part of putting the community’s vision into practice. Instead of “inefficient,” slowing down and paying attention (in this case, to microorganisms and vegetal energies) are framed as strategies for doing more with less, focused on producing better quality livelihoods on their own terms.

## Conclusion

This article has worked to trace how ecovillage residents attempt to make their respective communities “into a shape that cannot so easily be appropriated by a capitalist value system…[by] changing the frame of reference” (Odell 2019:23). By engaging with qualitative examples of how Rancho Bosque and Aldea Ceiba residents express understandings of *rentabilidad* in relation to more-than-human residents, I outline how changing terms (by embodying and performing alternative scales, values, and temporalities) articulates with changes in practice. In particular, these cases reveal how cultivating an expanded understanding of value in relation to daily practices of work helps ecovillage residents to make sense of and subvert latent interdependencies on broader capitalist systems.

These examples reveal paths forward in the sustainable development discourse, and suggest the generative possibility of revising the conceptual rubrics against which alternatives to development are compared. Insisting that ecovillage communities be formatted as “models” (which either succeed or do not) glosses over the complex daily negotiations that occur in the experimental and marginal spaces that seek to position themselves against the status quo (O’Hearn and Grubačić 2016). Removing the expectation that alternatives be “profitable” in a colloquial sense allows us to understand ecovillage projects as sites of productive experimentation, rather than scalable models. Instead, reflecting on the shifting, malleable relationships between time, capital, and value reveals how these relationships might be re-imagined or remade (Graeber 2001). What this article works towards instead is an understanding of the success of such projects on their own terms, rather than on capital-centric or growth-based rubrics. Rather, following Tsing (2015), I understand the language of scale to be incompatible with understanding alternatives in practice. Understanding how ecovillage residents grapple with these contesting currents is useful not because they provide models to replicate, but rather paths of resistance to follow.

## Footnotes

1 Pseudonyms are used for both communities, and some identifying details are deliberately obscured.

2 Morris (2022, in press) provides further discussion of multispecies ethnographic approaches employed elsewhere in the broader research on which this article is based.

3 Though *ejido* has been used as shorthand to describe all communally held lands in Mexico, two types of arrangements are subsumed under this label. While *ejidos* could be applied for by many sorts of communities or small groups, *comunidades* were particularly set aside for indigenous communities who were able to demonstrate an ancestral connection to the land in question. The conflation of the two systems into one categorical label is due in part to effects of colonialism; for instance, indigenous communities applied for *ejido* rights because of a lack of documentation of their indigenous status (c.f. Stephen 1994).

4 In two other communities consulted during this research period, disagreements over land ownership led to the departures of founding members.

5 *Melipona beecheii* do not have a stinger, and are generally not considered to be an aggressive species.

6 Traditional cultivation of *Melipona beecheii* is in widespread decline, as are wild populations of the insect (Villanueva-G and Roubik 2005).

7 The common names of each of these tree species are in Maya; *pich* is also referred to as *guanacaste* in Spanish.

8 The process of repetitively stirring preparations in water (“dynamization”) is a practice associated with biodynamic agriculture, the particular approach to which the ecovillage community had adopted as a guiding set of strategies for raising their livestock herds and cultivating their gardens.
